# Supplementary material for: Multi‐dataset identification of innovative feature genes and molecular mechanisms in keratoconus
Source: J Cell Mol Med. 2024 Sep 19;28(18):e70079. doi: 10.1111/jcmm.70079 (PMC11412914; doi:10.1111/jcmm.70079)
Supplement: Supplementary file 2 — Material S2. [file JCMM-28-e70079-s002.zip › 7.GSEA analysis/1.ARL11/KEGG_RETINOL_METABOLISM.html]

Details for gene set KEGG\_RETINOL\_METABOLISM[GSEA]

|  || Dataset | ARL11.group.cls#h\_versus\_l |
| Phenotype | group.cls#h\_versus\_l |
| Upregulated in class | h |
| GeneSet | KEGG\_RETINOL\_METABOLISM |
| Enrichment Score (ES) | 0.2694837 |
| Normalized Enrichment Score (NES) | 0.8717995 |
| Nominal p-value | 0.7338552 |
| FDR q-value | 1.0 |
| FWER p-Value | 1.0 |
Table: GSEA Results Summary

  

Fig 1: Enrichment plot: KEGG\_RETINOL\_METABOLISM      
 Profile of the Running ES Score & Positions of GeneSet Members on the Rank Ordered List

  

| SYMBOL | TITLE | RANK IN GENE LIST | RANK METRIC SCORE | RUNNING ES | CORE ENRICHMENT || 1 | RPE65 | na | 1088 | 0.378 | 0.0225 | Yes |
| 2 | UGT2B7 | na | 1178 | 0.368 | 0.0724 | Yes |
| 3 | UGT2B15 | na | 2914 | 0.244 | 0.0572 | Yes |
| 4 | UGT2B11 | na | 2923 | 0.244 | 0.0918 | Yes |
| 5 | CYP3A7 | na | 3477 | 0.220 | 0.1072 | Yes |
| 6 | UGT2B4 | na | 3576 | 0.216 | 0.1352 | Yes |
| 7 | ADH4 | na | 3617 | 0.215 | 0.1646 | Yes |
| 8 | UGT1A8 | na | 3762 | 0.209 | 0.1903 | Yes |
| 9 | CYP2C19 | na | 4519 | 0.182 | 0.1944 | Yes |
| 10 | UGT1A6 | na | 4803 | 0.172 | 0.2108 | Yes |
| 11 | CYP26C1 | na | 5252 | 0.159 | 0.2205 | Yes |
| 12 | UGT2B28 | na | 5683 | 0.146 | 0.2288 | Yes |
| 13 | CYP1A2 | na | 5834 | 0.141 | 0.2447 | Yes |
| 14 | ADH6 | na | 6053 | 0.136 | 0.2577 | Yes |
| 15 | CYP4A11 | na | 6286 | 0.129 | 0.2695 | Yes |
| 16 | CYP3A5 | na | 7636 | 0.099 | 0.2447 | No |
| 17 | BCO1 | na | 8073 | 0.091 | 0.2451 | No |
| 18 | CYP26B1 | na | 9524 | 0.067 | 0.2128 | No |
| 19 | UGT2B10 | na | 9893 | 0.062 | 0.2109 | No |
| 20 | PNPLA4 | na | 10992 | 0.048 | 0.1861 | No |
| 21 | DGAT1 | na | 11620 | 0.040 | 0.1737 | No |
| 22 | LRAT | na | 12132 | 0.035 | 0.1639 | No |
| 23 | CYP2B6 | na | 12739 | 0.029 | 0.1506 | No |
| 24 | UGT2A3 | na | 13634 | 0.022 | 0.1280 | No |
| 25 | CYP3A4 | na | 14527 | 0.015 | 0.1043 | No |
| 26 | DGAT2 | na | 14557 | 0.015 | 0.1056 | No |
| 27 | RETSAT | na | 15515 | 0.007 | 0.0790 | No |
| 28 | ADH1B | na | 16426 | 0.001 | 0.0529 | No |
| 29 | ALDH1A1 | na | 16456 | 0.001 | 0.0521 | No |
| 30 | CYP2C9 | na | 17344 | -0.006 | 0.0274 | No |
| 31 | RDH11 | na | 18137 | -0.011 | 0.0061 | No |
| 32 | RDH10 | na | 18366 | -0.013 | 0.0014 | No |
| 33 | CYP26A1 | na | 18525 | -0.014 | -0.0012 | No |
| 34 | ADH7 | na | 18569 | -0.014 | -0.0004 | No |
| 35 | CYP2C8 | na | 18873 | -0.016 | -0.0069 | No |
| 36 | RDH5 | na | 19471 | -0.020 | -0.0212 | No |
| 37 | CYP2C18 | na | 20676 | -0.029 | -0.0518 | No |
| 38 | UGT2A1 | na | 20689 | -0.029 | -0.0481 | No |
| 39 | CYP2A7 | na | 21232 | -0.033 | -0.0590 | No |
| 40 | ALDH1A2 | na | 21349 | -0.034 | -0.0575 | No |
| 41 | DHRS4L2 | na | 21609 | -0.036 | -0.0598 | No |
| 42 | DHRS4 | na | 21842 | -0.038 | -0.0611 | No |
| 43 | ADH5 | na | 23842 | -0.060 | -0.1102 | No |
| 44 | DHRS3 | na | 24128 | -0.064 | -0.1092 | No |
| 45 | CYP2A13 | na | 25078 | -0.077 | -0.1256 | No |
| 46 | RDH16 | na | 26897 | -0.106 | -0.1629 | No |
| 47 | CYP4A22 | na | 27781 | -0.125 | -0.1705 | No |
| 48 | DHRS9 | na | 29651 | -0.177 | -0.1992 | No |
| 49 | RDH12 | na | 30107 | -0.191 | -0.1851 | No |
| 50 | RDH8 | na | 31845 | -0.254 | -0.1989 | No |
| 51 | CYP3A43 | na | 32273 | -0.273 | -0.1723 | No |
| 52 | ADH1C | na | 33242 | -0.340 | -0.1517 | No |
| 53 | ADH1A | na | 33507 | -0.360 | -0.1080 | No |
| 54 | CYP1A1 | na | 34339 | -0.484 | -0.0630 | No |
| 55 | AWAT2 | na | 34464 | -0.520 | 0.0076 | No |
Table: GSEA details [plain text format]

  

Fig 2: KEGG\_RETINOL\_METABOLISM      
 Blue-Pink O' Gram in the Space of the Analyzed GeneSet

  

Fig 3: KEGG\_RETINOL\_METABOLISM: Random ES distribution      
 Gene set null distribution of ES for **KEGG\_RETINOL\_METABOLISM**
